# Meta-Dome for Broadband Radar Absorbing Structure

**DOI:** 10.1038/s41598-018-36273-8

**Published:** 2018-12-17

**Authors:** Heijun Jeong, Toan Trung Nguyen, Sungjoon Lim

**Affiliations:** School of Electrical and Electronic Engineering, Chung-Ang University, Heukseok-Dong Dongjak-Gu, 156-756 Republic of Korea

## Abstract

This study focused on the radome and proposed an absorber with a meta-dome structure for ultra-wideband radar absorption using an FR-4 dielectric material on the metasurface absorber for protection. In addition to protecting the absorber, the metasurface absorber exhibited ultra-wideband frequency absorptivity from radar signals, with an absorptivity band from 4.6–12 GHz, including the C and X frequency bands of radar signals. A wide incidence angle should also be considered in addition to the absorption frequency band. Experimental results were obtained for all polarization angles at normal incidence for 5–14 GHz. Sensitivity to incident angle from 0° to 40° in the transverse electric mode and 0° to 60° in the transverse magnetic mode were observed. The proposed concept was demonstrated using full-wave simulation and experimental measurements.

## Introduction

Radome is a compound word for a radar dome, a device that maintains radar performance and protects the radar from the external environment^1,2^. For example, since aircraft move at high speeds, it is essential to protect the radar from wind pressure, lightning, hail, etc. For these radome technologies^3,4^, dielectric materials are commonly used that can protect while maintaining the characteristics of the radar^5,6^. In addition, the dielectric materials can be used to maintain the impedance matching for wide incident angle which is called as the wide-angle impedance matching (WAIM)^7^. The WAIM techniques have been mostly applied in planar array antennas by loading the dielectric constant sheet^7^, dielectric slab^8,9^, or frequency selective surface (FSS)^10–14^ on the top of the antenna array. When the WAIM concept is applied to electromagnetic absorbers, wide incidence angle and wide bandwidth absorption can be achieved.

**Figure 1:**
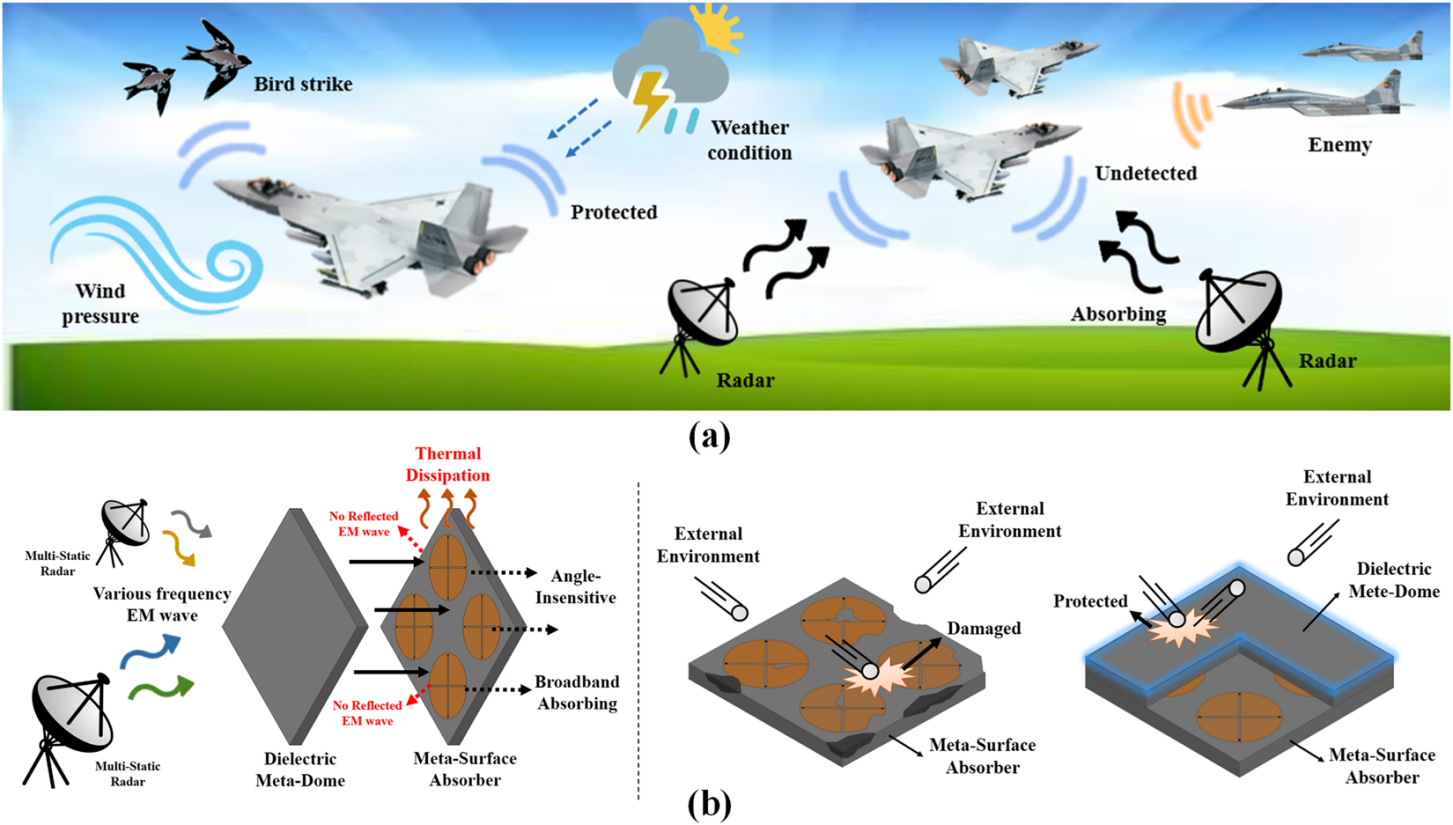
Proposed meta-dome concept: (**a**) motivation and (**b**) structure roles as absorbers and meta-domes.

Figure 1 shows the concept of the proposed meta-dome application. Metamaterial absorber applications used in stealth technology should be protected from external environments, such as wind pressure, bird strike, and weather conditions, as well as be undetectable by radar signals. Hence, the radar-absorbing structures should be outermost. However, radar-absorbing structures can be significantly affected by the external environment. The proposed technology also offers ultra-wideband radar absorption to protect the radar-absorbing structure. Metasurfaces were first proposed by Landy^15^ as radar absorbing structures. A metasurface is a structure where an infinite number of artificial structures are periodically arranged. These metasurfaces can control material permittivity and permeability, and various metasurfaces characteristics can be applied to electromagnetic absorbers such as stealth technologies^16–18^, sound waves^19–21^, human body^22^, super lenses^23,24^, terahertz applications^25–27^, and electromagnetic interface (EMI)/electromagnetic compatibility (EMC) solutions. Metasurface based absorbers have advantages over materials based absorbers, such as wedge tapered absorbers^28–30^ and Jaumann absorbers^31,32^. Wedge tapered absorbers have excellent absorptivity, but are bulky and ferrite used is an expensive material.

To overcome these drawbacks, Jaumann absorbers use quarter wavelength (λ/4) thickness of dielectric materials. Although one can make thinner wedge tapered absorbers, they remain quite bulky since they must be λ/4 thick. In contrast, hand, metasurface absorbers are based on LC resonance and thus not affected by material thickness. Consequently, metasurface absorbers exhibit high absorptivity, thin structure, and are easy to fabricate; cost-effective.

Despite these metasurface absorber advantages, they have the disadvantage of narrow absorptivity bandwidth because they use LC resonance for absorptivity. Broadband absorbers are required for practical applications radar signals and comprise several frequency bands, hence metasurface absorbers should also have broadband absorptivity to absorbing the various radar signals. Several methods have been proposed to construct broadband metasurface absorbers, including multiple resonance^33–35^, resistive ink or pattern^36–38^, and lumped elements^39,40^.

This study adopted lumped elements for the broadband radar-absorbing structure, since they, e.g. the chip resistor method, provide excellent broadband absorptivity and are also easily applied on printed circuit boards. in addition to broadband absorptivity, modern radar technologies use multi-static signals to detect objects, as shown Fig. 1(b). Metasurface absorbers must be angle insensitivity to absorb multiple angle radar signals. Most wide incidence angle metamaterial absorbers have been realized using angle insensitive unit cell designs, such as the split ring cross resonator^41^, circular sector^42,43^ and surrounding via array^44^, subwavelength unit cell^45^, and four-fold rotational symmetric electric resonator structure^46^. Wide incidence angle absorption can be achieved by loading a dielectric material on the metamaterial absorber, due to WAIM, which also increases the absorption bandwidth due to the high loss of WAIM.

This study proposes a meta-dome to not only enhance broadband radar-absorbing structure and, but also to protect the structure from the external environment. For example, meta-domes are placed on radar-absorbing structures to protect them from mechanical damage. The proposed meta-dome structure has broadband absorptivity in addition to its protecting properties. We also propose a polarization and incidence angle insensitive structure for both transverse electric (TE) and transverse magnetic (TM) modes. Performance of the proposed structure was demonstrated through full wave simulations and measurements. In particular, we studied the effects of the dielectric materials on the top of the metasurface by comparing absorptivity of the metamaterial absorber with and without the dielectric material.

## Numerical Simulations

**Figure 2:**
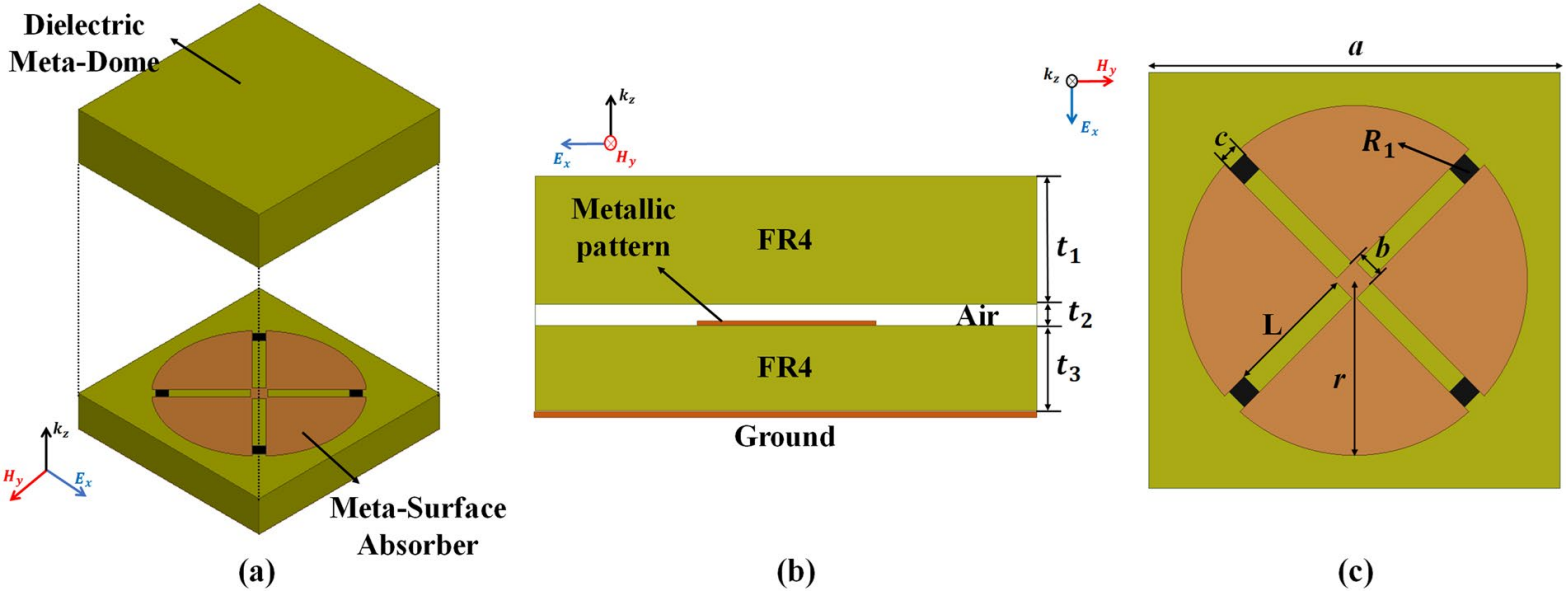
Proposed meta-dome unit cell geometry: (**a**) perspective, (**b**) side, and (**c**) top view. Conductive pattern, substrate, and chip resistors are shown in ocher, green, and black, respectively.

Figure 2 shows the unit cell geometry for the proposed meta-dome structure. The proposed meta-dome consists of a metasurface absorber and a dielectric meta-dome as shown Fig. 2(a). We placed the dielectric meta-dome on the metasurface absorber to protect it and used fire retardant or flame resistant 4 (FR4) substrates (dielectric constant *ɛ*_*r*_ = 4.4 and loss tangent = 0.02) for the metasurface absorber and meta-dome, as shown Fig. 2(b). Substrate thicknesses *t*_1_ = *t*_3_ = 2.4 mm, and we employed a fan shaped structure to achieve electromagnetic resonance, as in shown Fig. 2(c). Since the proposed shape was symmetrical, the meta-dome is polarization and angle insensitive. The conductive pattern dimensions were *a = *14 mm, L = 3.02 mm, *r* = 4 mm, *b* = 0.5 mm, and *c* = 0.35 mm. For broadband absorption, we used chip resistors (R_1_ = 150 Ω). It is impossible to avoid air gaps when the metasurface absorber and dielectric meta-dome are joined, hence we set an air gap *t*_2_ = 0.4 mm. We employed the ANSYS high frequency structure simulator (HFSS) for numerical simulations.

Absorptivity can be expressed as

1 $$A(\omega )=1-{\rm{\Gamma }}(\omega )-T(\omega ),$$

where Γ(ω) and T(ω) are the reflection and transmission coefficients, respectively. Since the bottom ground plane of the proposed meta-dome was completely covered with copper, we can set T(ω) = 0, and the highest absorptivity will be achieved when Γ(ω) = 0.

For normal incidence,

2 $${\rm{\Gamma }}({\rm{\omega }})=\frac{{{\rm{Z}}}_{{\rm{0}}}-{{\rm{Z}}}_{M}}{{{\rm{Z}}}_{{\rm{0}}}+{{\rm{Z}}}_{M}},$$

where Z_0_ = 377 Ω and Z_M_ are free space and metasurface impedance, respectively. Therefore, we can achieve maximum absorptivity when Z_M_ = Z_0_, i.e., Γ(ω) = 0. We can express Z_M_ as

3 $${{\rm{Z}}}_{M}=\sqrt{\frac{{\mu }_{{\rm{0}}}{\mu }_{r}}{{\varepsilon }_{{\rm{0}}}{\varepsilon }_{r}}},$$

where μ_r_ is the effective permeability, and ɛ_r_ is the effective permittivity. Therefore, impedance matching occurs between Z_M_ and Z_0_ when μ_r_ = ɛ_r_.

However, for oblique incidence, absorptivity decreases as the incidence angle increases, due to the wave amplitude reflected from the metasurface absorber varying. We can express the reflection coefficients for perpendicular (Z_⊥_) and parallel (Z_∥_) polarization for oblique incidence as

4 $${Z}_{\perp }=\frac{{Z}_{M}\,\cos \,{\theta }_{i}-{Z}_{0}\,\cos \,{\theta }_{t}}{{Z}_{M}\,\cos \,{\theta }_{i}+{Z}_{0}\,\cos \,{\theta }_{t}}$$

and

5 $${Z}_{||}=\frac{{Z}_{M}\,\cos \,{\theta }_{t}-{Z}_{0}\,\cos \,{\theta }_{i}}{{Z}_{M}\,\cos \,{\theta }_{t}+{Z}_{0}\,\cos \,{\theta }_{i}},$$

where *θ*_*i*_ and *θ*_*t*_ are the incidence and transmission angles, respectively. Therefore, we should consider an angle insensitive metasurface unit cell^38^ to achieve maximum absorptivity for oblique incidence.

**Figure 3:**
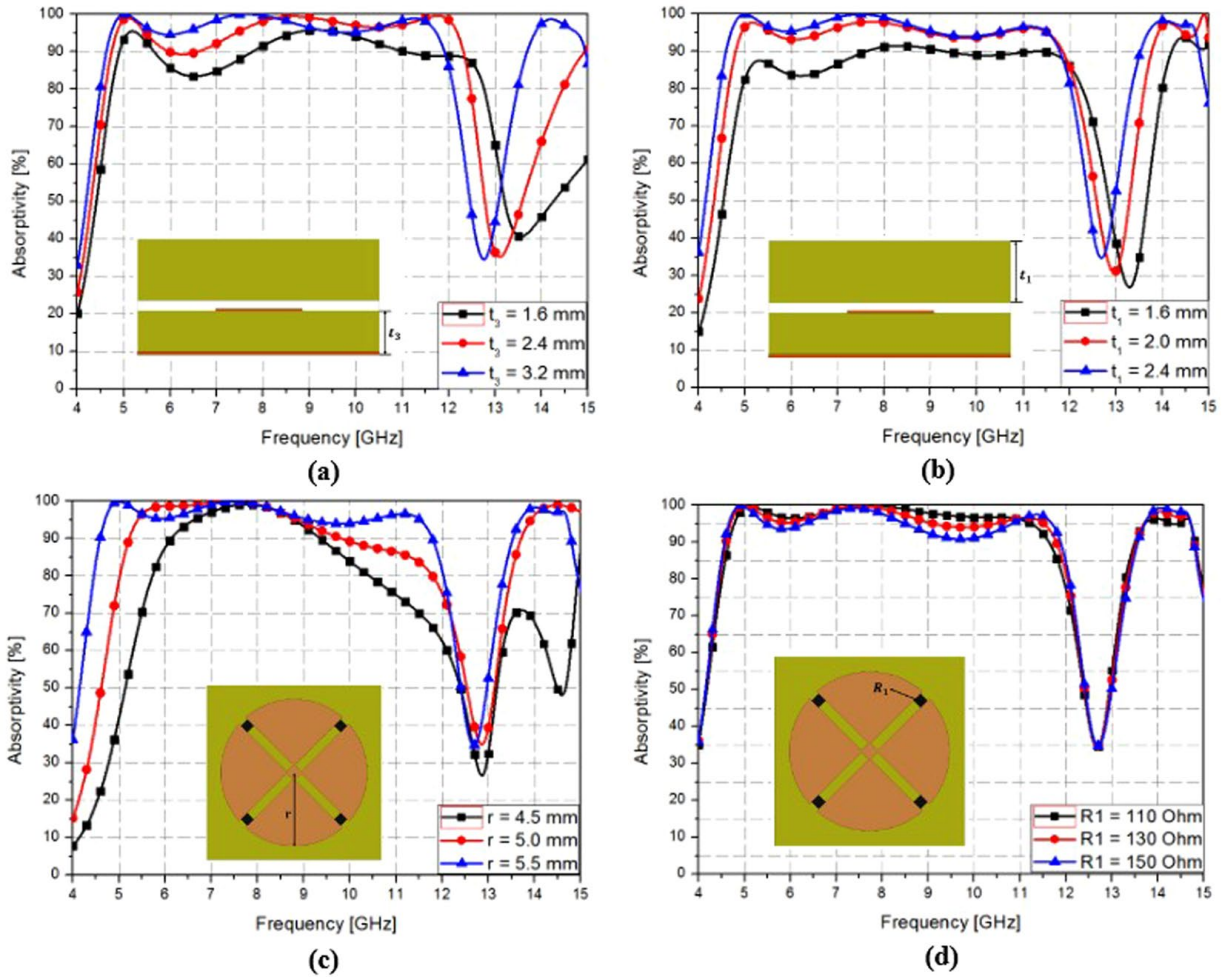
Simulated absorptivity for the proposed meta-dome for different (**a**) metasurface absorber substrate thickness (t_3_), (**b**) meta-dome substrate thickness (t_1_), (**c**) conductive pattern (*r*), and (**d**) chip resistor values (R_1_).

Figure 3 shows simulated absorptivity for various parameter values. When metasurface absorber substrate thickness t_3_ varied from 1.6–3.2 mm, absorptivity increased as *t*_3_ increased. However, absorbance bandwidth decreased as *t*_3_ increased. Therefore, we set *t*_3_ = 2.4 mm, as shown Fig. 3(a). Similarly, absorptivity increased as meta-dome substrate *t*_1_ varied from 1.6–2.4 mm, as shown in Fig. 3(b). We also set *t*_1_ = 2.4 mm since absorptivity saturated above 2.4 mm. We set radome radius *r* = 5.5 mm since that offered maximum absorption bandwidth (see Fig. 3 (c)). For broadband absorptivity, we used a chip resistor with best absorptivity at 150 Ω, as shown Fig. 3(d).

**Figure 4:**
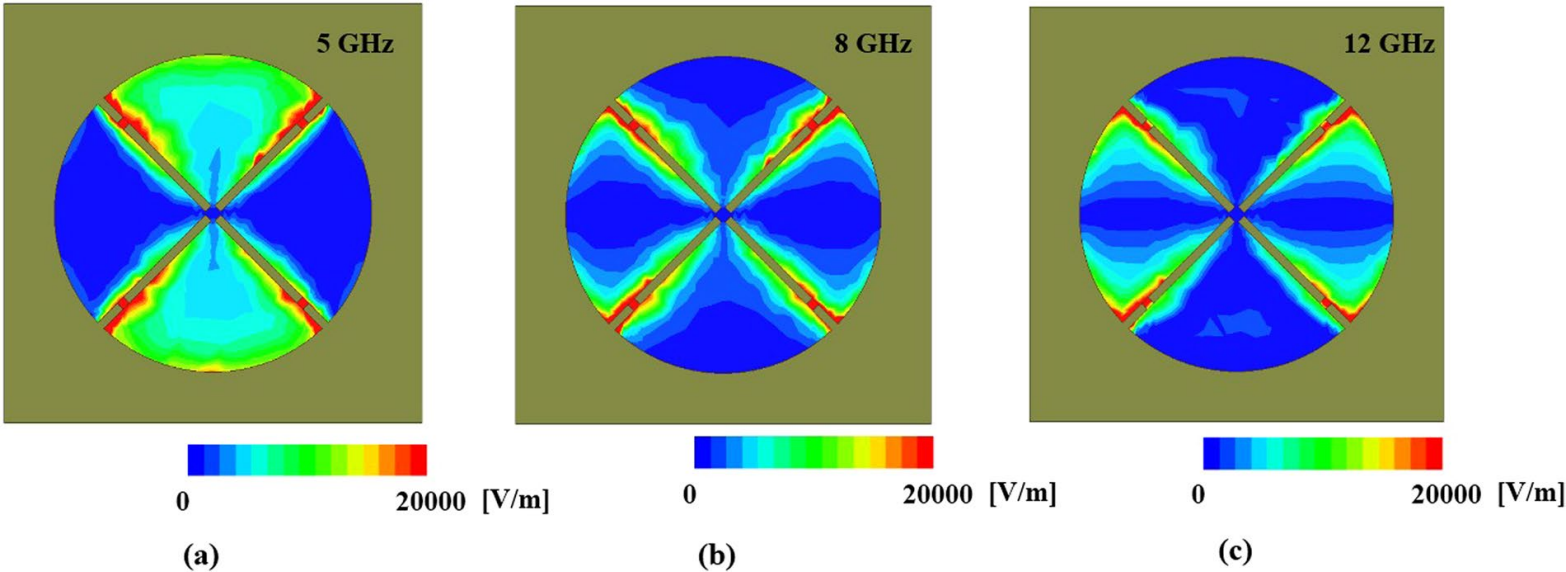
Simulated electric field distribution for the proposed meta-dome unit cell at (**a**) 5, (**b**) 8, and (**c**) 12 GHz.

Figure 4 shows the simulated electric field distribution for the proposed meta-dome unit cell. At 5 GHz, the electric field was distributed between the top and bottom sectors of the metasurface patterns and near the chip resistors, as shown in Fig. 4(a). Similarly, at 8 and 12 GHz, the electric field was distributed between the right and left sectors of the metasurface patterns and near the chip resistors, as shown in Fig. 4(b,c), respectively. Consequently, electromagnetic resonance arises from each sector, and the resonance frequencies constitute the absorptivity frequencies. The chip resistors also produce strong electric fields that consume energy near the electromagnetic resonance frequencies. Therefore, we can achieve broadband absorptivity using chip resistors.

**Figure 5:**
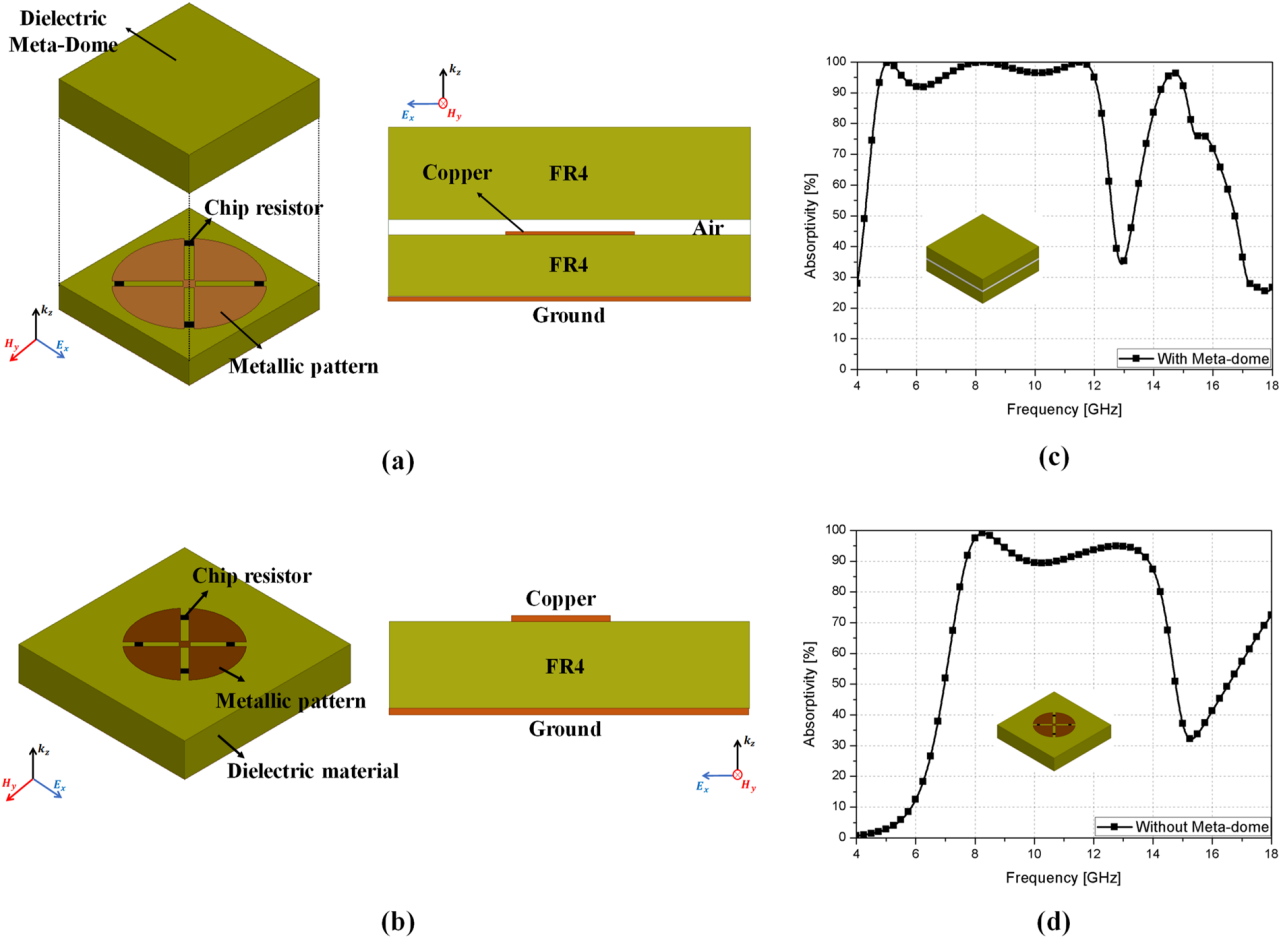
Metasurface structure (**a**) with and (**b**) without dielectric material; and simulated absorptivity (**c**) with and (**d**) without dielectric material.

To investigate the effect of the dielectric material above the metasurface, absorptivity under normal incidence was simulated with and without the dielectric material, as shown in Fig. 5(a,b), respectively, and simulated absorptivity in Fig. 5(c,d). The metasurface without dielectric material (Fig. 5(c)) has more than 90% absorptivity from 7.8–13.9 GHz, corresponding to 56% bandwidth. On the other hand, the metasurface with dielectric material (Fig. 5(d)) has more than 90% absorptivity from 4.6–12.2 GHz, corresponding to 90% bandwidth. Therefore, wider bandwidth was achieved by using higher loss material.

**Figure 6:**
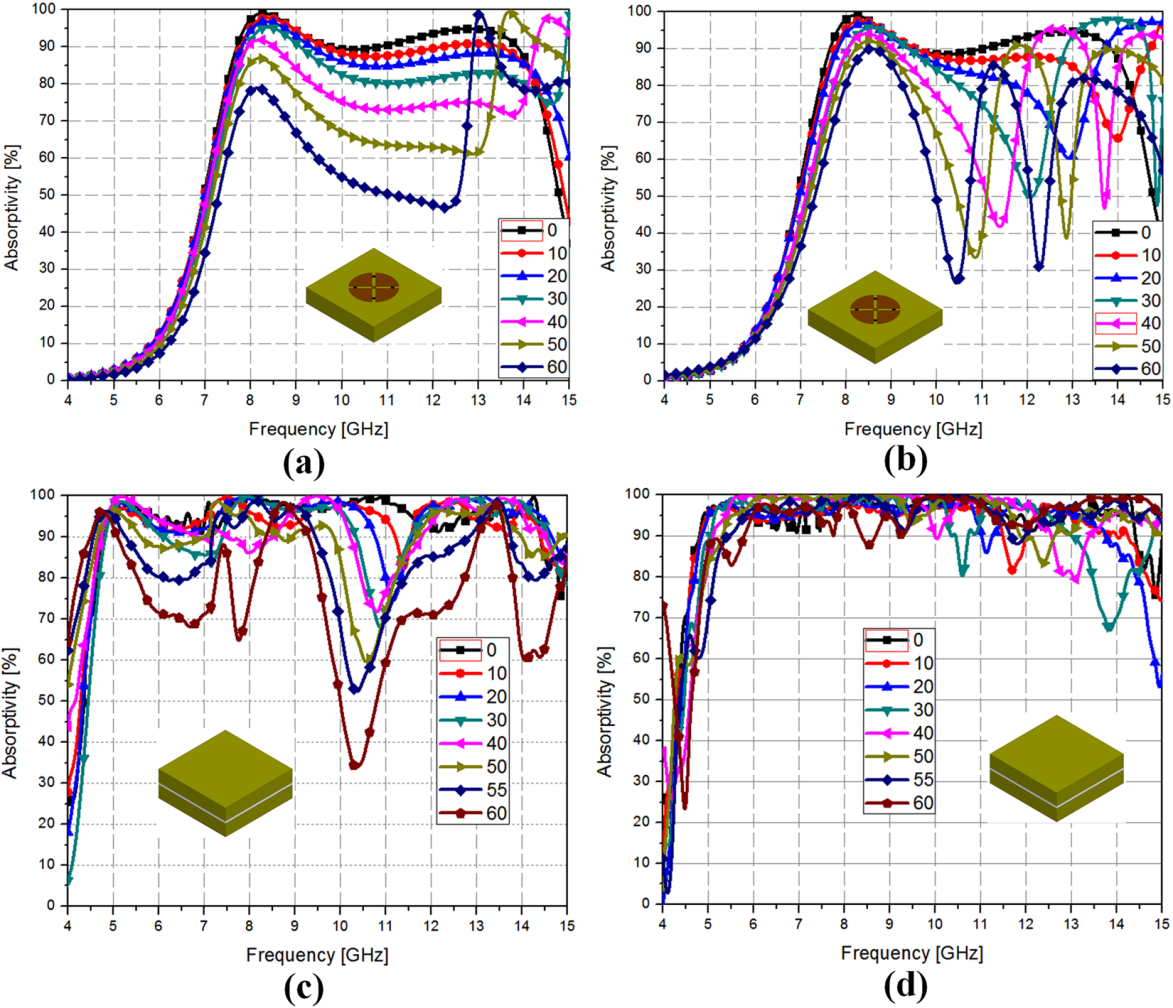
Metasurface simulated absorptivity for oblique incidence without dielectric material for (**a**) TE and (**b**) TM mode; and with dielectric material for (**c**) TE and (**d**) TM mode.

Figure 6 shows simulated WAIM effects for the proposed meta-dome under oblique incidence from 0° to 60°with and without dielectric material. For normal incidence, the metamaterial absorber without dielectric material exhibited more than 90% absorptivity from 7.8–13.9 GHz, which decreased to 7.8–8.5 GHz and 8–9 GHz for TE and TM mode at 40° incidence, as shown Fig. 6(a,b), respectively. On the other hand, metamaterial absorber with dielectric material exhibited more than 90% absorptivity from 4.8–14.4 GHz under normal incidence, which only slightly decreased to 4.8–10.4 GHz in TE mode, as shown in Fig. 6(c); and was maintained at 4.8–14.4 GHz in TE mode up to 60° incidence, as shown Fig. 6(d). Table 1 compares 90% absorption bandwidth under different oblique incidence with and without the dielectric material. Thus, the proposed meta-dome absorber achieved wide incidence absorption.

**Table 1:** Metasurface 90% absorption bandwidth with and without dielectric material under normal and oblique incidence.

Polarization	Incident Angle (°)	90% Absorptivity with dielectric material		90% Absorptivity without dielectric material	
		Frequency (GHz)	BW^a^ (%)	Frequency (GHz)	BW^a^ (%)
TE	0	4.6–12.2	7.6	7.8–13.9	6.1
	10	4.6–11.3	6.7	7.8–13.5	5.7
	20	4.6–10.8	6.2	7.8–9.5	1.7
	30	4.6–10.4	5.8	7.8–9.0	1.2
	40	4.6–10.4	5.8	7.8–8.5	0.7
TM	0	4.6–12.2	7.6	7.8–13.9	6.1
	10	4.6–12.2	7.6	7.8–9.5	1.7
	20	4.6–12.2	7.6	7.8–9.5	1.7
	30	4.6–12.2	7.6	7.8–9.5	1.7
	40	4.6–12.2	7.6	8.0–9.0	1.0
	50	4.6–12.2	7.6	N/A	N/A
	60	4.6–12.2	7.6	N/A	N/A

^a^$$BW={\rm{\Delta }}f/{f}_{c},where\,{\rm{\Delta }}f={f}_{high}-{f}_{low}\,and\,{f}_{c}=({f}_{high}+{f}_{low})/2$$.

## Experimental Verification

**Figure 7:**
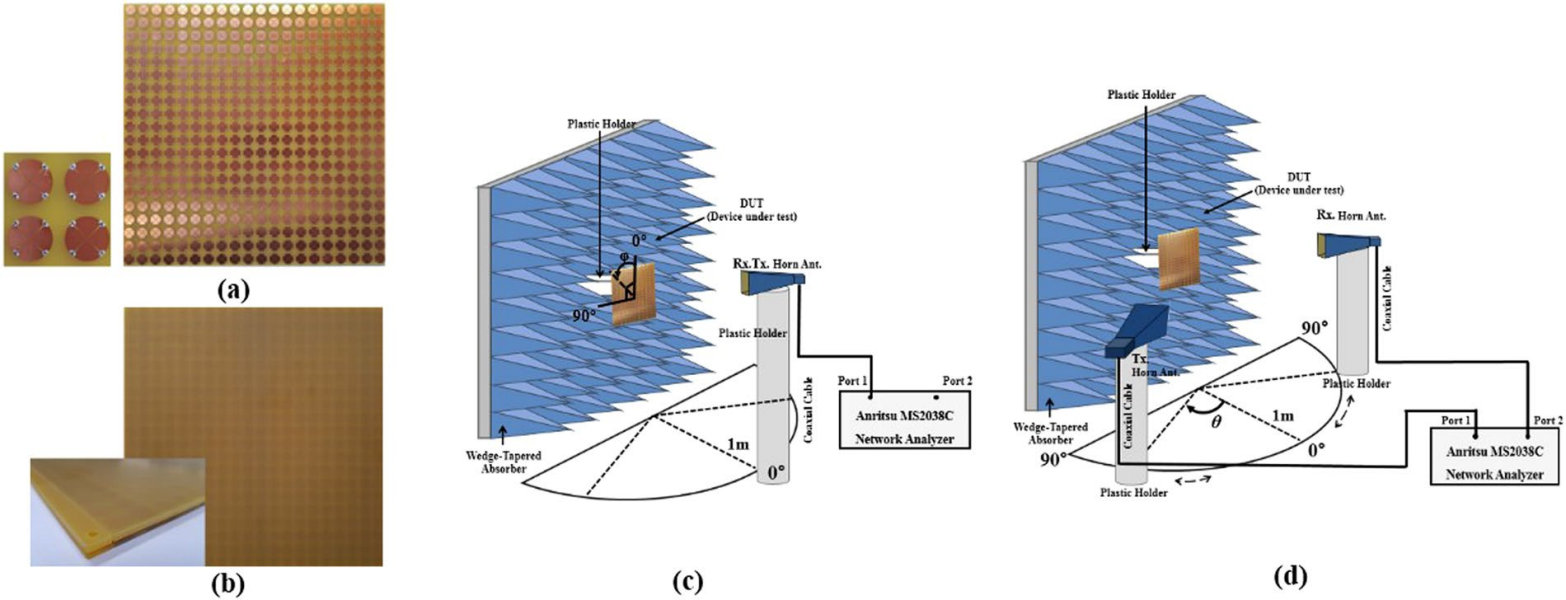
Proposed meta-dome experimental verification: fabricated (**a**) metasurface absorber top view, (**b**) meta-dome structure top view; and (**c**) mono-static and (**d**) bi-state environment measurement setup.

To experimentally demonstrate the meta-dome structure, we constructed prototype sample 280 × 280 mm, consisting of 20 × 20 unit cells. The metasurface substrate comprised top conductive patterns and bottom ground plane, as shown Fig. 7(a). The top conductive patterns consisted of copper fan shaped patterns and 1.6 × 0.8 mm chip resistors (1608 metric code). Each chip resistor has a resistance of 150 Ω, mounted by surface mount technology (SMT) processing. The meta-dome substrate contained only dielectric materials without any conductive patterns. Overall size of the fabricated meta-dome substrate = 280 × 280 mm, the same as the metasurface substrate, as shown Fig. 7(b).

We set up two environments to measure the prototype samples. Figure 7(c) shows the mono-static measurement environment. Since the bottom plane of the proposed meta-dome was totally covered with metallic ground, transmission = zero. Therefore, we only needed to measure the reflection coefficient from the sample. We placed wedge tapered absorbers behind the sample to prevent unexpected reflection signals other than those from the sample, and measured the reflected signal from the sample only using the time gating method. We set distance between the sample and antenna = 1 m to satisfy the far-field condition, and measured the reflection coefficient using a Dorado AN-GH1-18N horn antenna (frequency range 1–18 GHz) and Anritsu MS2038 vector network analyzer (VNA). Calibration was achieved by setting the reflection coefficient with the copper plate to 0 dB before measuring the S parameters. To demonstrate polarization insensitivity, we measured the reflection coefficient for different polarization angles by rotating the sample from 0° to 90°.

We set up the bi-state measurement environment shown in Fig. 7(d) to demonstrate angle insensitivity. We employed two Dorado AN-GH1-18N horn antennas and Anritsu MS2038 VNAs using the two-port measurement method. To measure both TE and TM modes, we rotated the antenna 0° and 90° and placed the antennas at different incident angles from 0° to 60°.

**Figure 8:**
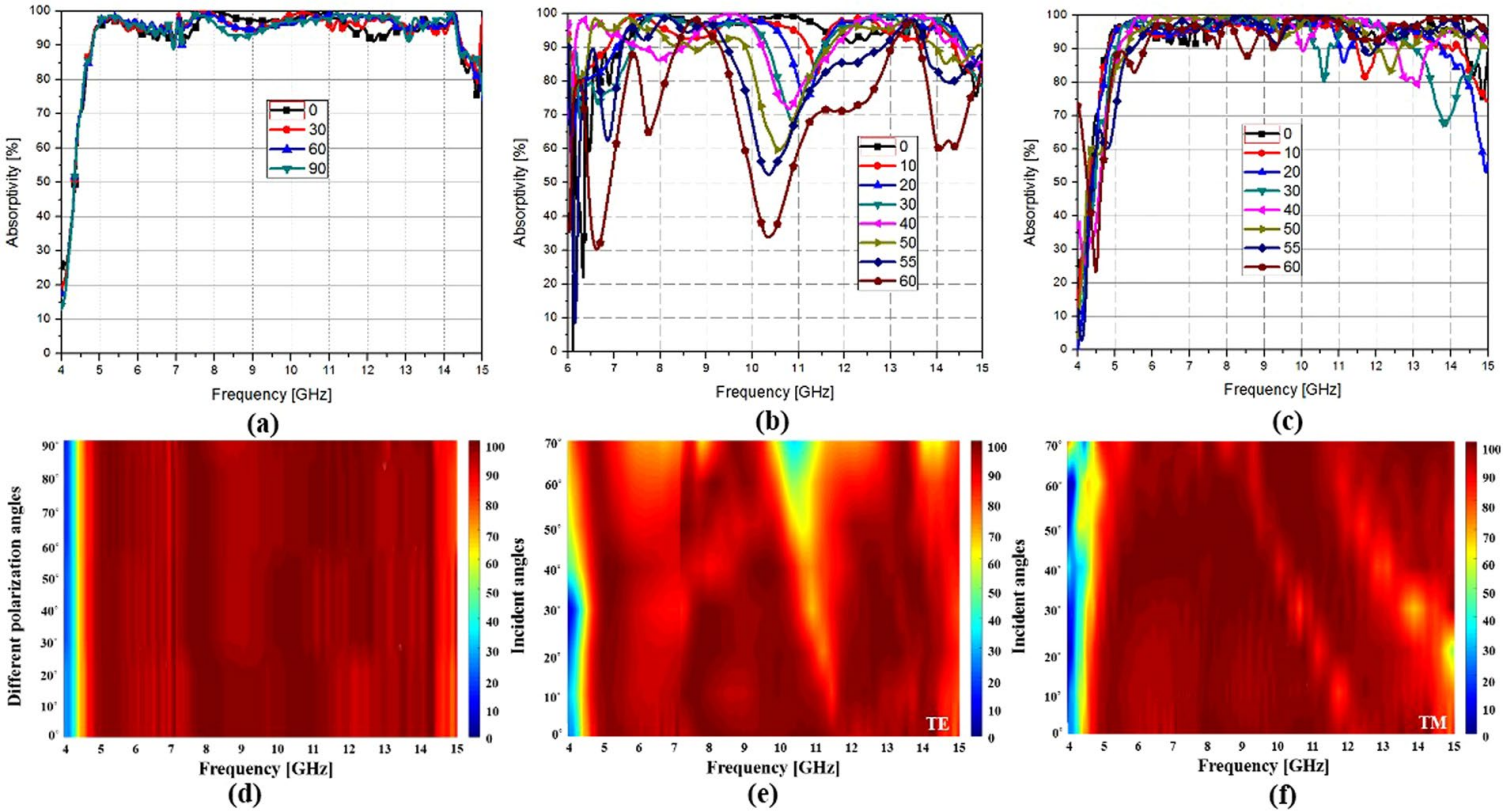
Proposed meta-dome prototype measurement results for (**a**) normal incidence; (**b**) oblique incidence, TE mode; (**c**) oblique incidence, TM mode; and specular angle of the proposed meta-dome for (**d**) different polarization angles, and oblique incidence (**e**) TE and (**f**) TM mode.

Figure 8 shows the measurement results from equation (1) for the proposed meta-dome. Under normal incidence, 90% absorptivity bandwidth was observed between 4.8–14.4 GHz. Since the proposed meta-dome was symmetrical, we measured the absorptivity bandwidth at polarization from 0°–90°, as shown in Fig. 8(a), and polarization insensitivity is evident from Fig. 8(d).

Figure 8(b,e) show measurement results for oblique incidence TE mode. Comparing Fig. 8(b,e), the proposed meta-dome absorptivity was similar to simulation outcomes, except between 0°–40° for TE mode. However, measured absorptivity was very similar to the simulation results from 0°–60° for TM mode as shown in Fig. 8(c,f). Thus, we confirmed that normal incidence was insensitive to 0°–90° polarization within 4.8–14.4 GHz, and oblique incidence was insensitive to 0°–40° and 0°–60° incidence angle in TE and TM mode, respectively. Therefore, the proposed meta-dome showed excellent agreement between the fabricated prototype and simulation outcomes.

## Methods

### Numerical Simulations

We used the ANSYS high frequency structure simulator (HFSS), a full-wave simulator, to perform simulations. After designing the unit cell, we set two pairs as master and slave and assumed an infinite periodic structure, assigning two XZ, YZ planes as master/slave pairs. Two floquet ports were used as excitation ports. The air box was 14 × 14 × 15 mm. The copper used for the conductive pattern had resistivity = 58 × 10^6^ S/m.

### Experimental Verification

To perform the experimental measurements, we used the setup for normal and oblique incidences in free space, and placed wedge tapered absorbers behind the sample. to prevent unexpected signal reflections. Since the bottom plate of sample was completely covered with copper ground, we measured only the reflection coefficient, using two DORADO AN-GH1-18N devices (frequency 1–18 GHz) horn antennas and an ANRITS MS2038 vector network analyzer (VNA). We used the time-gating method to measure only signals reflected from the sample. To satisfy the far-field condition, we set the distance between the sample and horn antennas = 1 m. For normal incidence, we used a horn antenna, measuring each polarization 0°–90° each 10°; and measured TE and TM for oblique incidence using two horn antennas from 0°–60° incidence every 10°.

## Discussion

Broadband absorptivity is required for modern stealth technology applications, because radar signals are detected through multiplexed frequency bands. This paper proposed a meta-dome structure for ultra-wideband radar absorption, and we placed dielectric materials on the metasurface absorber o protect the metasurface absorber from the external environment, and incorporated fan shaped conductive patterns in the metasurface absorbers. The proposed radar antenna and radome achieved broadband absorptivity with chip resistors and demonstrated polarization and angle insensitivity when absorbing multi-static radar signals.

We simulated the proposed device using a full-wave simulation tool to demonstrate operational performance, and fabricated a 280 × 280 mm sample of 20 × 20 unit cells. The fan-shaped conductive patterns were fabricated using PCB etching, and employed SMT chip resistors. Mono and bi-static measurements stations were setup for experimental validation. We used horn antennas and VNAs to measure normal incidence, and a time-gating method to measure the reflected signals. Two horn antennas and a VNA were employed to measure oblique incidence incorporating time-gating to obtain TE and TM mode outcomes simultaneously.

Consequently, we confirmed that normal incidence was insensitive to 0°–90° polarization with 90% absorptivity for 4.8–14.4 GHz. The proposed meta-dome was angle insensitive 0°–40° for oblique incidence in TE mode and 0°–60° in TM mode.

Thus, the proposed meta-dome successfully improved radar detection demonstrated through full-wave simulations and experimental verification.

## References

[1] Persson KM, Gustafsson M, Kristensson G (2008). Reconstruction and visualization of equivalent currents on a radome using an integral representation formulation. Prog. Electromagn. Res. B.

[2] Chen F, Shen Q, Zhang L (2010). Electromagnetic Optimal Design and Preparation of Broadband Ceramic Radome Material with Graded Porous Structure. Prog Electromagn. Res. B.

[3] Haris MY (2011). Preliminary Review of Biocomposites Materials for Aircraft Radome Application. Key Eng. Mater..

[4] Peng M, Xian QL, Jai WY, Peng CZ, Abdelheq B (2018). A Low Radar Cross Section and Low Profile Antenna Co-Designed With Absorbent Frequency Selective Radome. IEEE Trans Antennas Propag..

[5] Barta J, Manela M, Fischer R (1985). Si3N4 and Si2N2O for high performance radomes. Mater. Sci. Eng..

[6] Sukharevsky OI, Vasilets VA (2008). Scattering of reflector antenna with conic *dielectric radom*e. Prog. Electromagn. Res. B.

[7] Magill EG, Wheeler HA (1966). Wide-angle impedance matching of a planar array antenna by a dielectric sheet. IEEE Trans. Antennas Propag..

[8] Galindo V, Wu CP (1968). Dielectric Loaded and Covered Rectangular Waveguide Phased Arrays. Bell Syst. Tech. J..

[9] Lee SW, Mittra R (1968). Radiation from Dielectric-Loaded Arrays of Parallel-Plate Waveguides. IEEE Trans. Antennas Propag..

[10] Bah AO, Qin PY, Ziolkowski RW, Cheng Q, Guo YJ (2018). Realization of an Ultra-thin Metasurface to Facilitate Wide Bandwidth, Wide Angle Beam Scanning. Sci. Rep..

[11] Cameron TR, Eleftheriades GV (2015). Analysis and Characterization of a Wide-Angle Impedance Matching Metasurface for Dipole Phased Arrays. IEEE Trans. Antennas Propag..

[12] Munk BA, Kouyoumjian RG, Peters L (1971). Reflection Properties of Periodic Surfaces of Loaded Dipoles. IEEE Trans. Antennas Propag..

[13] Hill RA, Munk BA (1996). The effect of Perturbating a Frequency Selective Surface and Its Relation to the Design of a Dual-Band Surface. IEEE Trans. Antennas Propag..

[14] Luebbers RJ, Munk BA (1978). Some Effects of Dielectric Loading on Periodic Slot Arrays. IEEE Trans. Antennas Propag.

[15] Landy NI, Sajuyigbe S, Mock JJ, Smith DR, Padilla WJ (2008). Perfect metamaterial absorber. Phys. Rev. Lett..

[16] Schurig D (2006). Metamaterial electromagnetic cloak at microwave frequencies. Science.

[17] Iwaszczuk K (2012). Flexible metamaterial absorbers for stealth applications at terahertz frequencies. Opt. Express..

[18] Wang FW, Gong SX, Zhang S, Mu X, Hong T (2011). RCS reduction of array antennas with radar absorbing structures. J. Electromagn. Waves Appl..

[19] Yang Z, Dai MH, Chan HN, Ma CG, Sheng P (2010). Acoustic metamaterial panels for sound attenuation in the 50-1000 Hz regime. Appl. Phys. Lett..

[20] Sebastien G, Alexander M, Gunnar P, Anantha SR (2007). Acoustic metamaterials for sound focusing and confinement. New J. Phys..

[21] Yifan Z (2018). Fine manipulation of sound via lossy metamaterials with independent and arbitrary reflection amplitude and phase. Nat. Commun..

[22] Jeon J, Lee S, Choi J, Kim S (2015). Analysis of Absorption Loss by Human Body in On-to-off Body Communication at 2.45 GHz. J. Electromagn. Eng. Sci..

[23] Newsome WT (2005). Sub-diffraction-limited optical imaging with a silver superlens. Science.

[24] Aydin K, Bulu I, Ozbay E (2007). Subwavelength resolution with a negative-index metamaterial superlens. Appl. Phys. Lett..

[25] Lee D, Sung H, Lim S (2016). Flexible subterahertz metamaterial absorber fabrication using inkjet printing technology. J. Phys. B.

[26] Landy N (2009). Design, theory, and measurement of a polarization-insensitive absorber for terahertz imaging. Phys. Rev. B.

[27] Tao H (2010). A dual band terahertz metamaterial absorber. J. Phys. D.

[28] Park MJ, Choi J, Kim SS (2000). Wide bandwidth pyramidal absorbers of granular ferrite and carbonyl iron powders. IEEE Trans. Magn..

[29] Shin JY, Oh JH (1993). The microwave absorbing phenomena of ferrite microwave absorbers. IEEE Trans. Magn..

[30] Kim D-Y, Yoon Y-H, Jo K-J, Jung G-B, An C-C (2016). Effects of sheet thickness on the electromagnetic wave absorbing characterization of Li0.375Ni0.375Zn0.25-ferrite composite as a radiation absorbent material. J. Electromagn. Eng. Sci..

[31] Toit LJD (1994). The design of Jauman absorbers. IEEE Antennas Propag. Mag..

[32] Fang X, Zhao CY, Bao H (2018). Design and analysis of Salisbury screens and Jaumann absorbers for solar radiation absorption. Front. Energy.

[33] Zhang H-B (2013). Resistance selection of high impedance surface absorbers for perfect and broadband absorption. IEEE Trans. Antennas Propag..

[34] Costa F, Monorchio A (2012). A frequency selective radome with wideband absorbing properties. IEEE Trans. Antennas Propag..

[35] Lee J, Lee B (2016). Design of thin RC absorbers using a silver nanowire resistive screen. J. Electromagn. Eng. Sci..

[36] Luo H, Hu X, Qiu Y, Zhou P (2014). Design of a wide-band nearly perfect absorber based on multi-resonance with square patch. Solid State Commun..

[37] Park JW (2013). Multi-band metamaterial absorber based on the arrangement of donut-type resonators. Opt. Express.

[38] Peng X-Y, Wang B, Lai S, Zhang DH, Teng J-H (2012). Ultra multi-band planar metamaterial absorber based on standing wave resonances. Opt. Express.

[39] Mias C, Yap JHA (2007). Varactor-tunable high impedance surface with a resistvie-lumped-element biasing grid. IEEE Trans. Antennas Propag..

[40] Costa F, Monorchio A, Manara G (2010). Analysis and design of ultra thin electromagnetic absorbers comprising resistively loaded high impedance surfaces. IEEE Trans. Antennas Propag..

[41] Cheng Y, Yang H, Cheng Z, Wu N (2011). Perfect metamaterial absorber based on a split-ring-cross resonator. Appl. Phys. A Mater. Sci. Process..

[42] Lee D, Hwang JG, Lim D, Hara T, Lim S (2016). Incident Angle- and Polarization- Insensitive Metamaterial Absorber using Circular Sectors. Sci. Rep..

[43] Nguyen TT, Lim S (2017). Wide Incidence Angle-Insensitive Metamaterial Absorber for Both TE and TM Polarization using Eight-Circular-Sector. Sci. Rep..

[44] Lim D, Lee D, Lim S (2016). Angle- and polarization-insensitive metamaterial absorber using via array. Sci. Rep..

[45] Yoo M, Kim HK, Lim S (2016). Angular- and polarization-insensitive metamaterial absorber using subwavelength unit cell in multilayer technology. IEEE Antennas Wirel. Propag. Lett..

[46] Zhu B (2010). Polarization insensitive metamaterial absorber with wide incident angle. Prog. Electromagn. Res..

